# Risk of serious COVID-19 outcomes among adults with asthma in Scotland: a national incident cohort study

**DOI:** 10.1016/S2213-2600(21)00543-9

**Published:** 2022-04

**Authors:** Ting Shi, Jiafeng Pan, Eleftheria Vasileiou, Chris Robertson, Aziz Sheikh, Public Health Scotland and the EAVE II Collaborators

**Affiliations:** a Usher Institute, Edinburgh Medical School, University of Edinburgh, Edinburgh, UK; b Department of Mathematics and Statistics, University of Strathclyde, Glasgow, UK; c Public Health Scotland, Glasgow, UK; d Asthma UK Centre for Applied Research, Usher Institute, University of Edinburgh, Edinburgh, Scotland, UK

## Abstract

**Background:**

There is considerable uncertainty over whether adults with asthma should be offered booster vaccines against SARS-CoV-2 and, if so, who should be prioritised for booster vaccination. We were asked by the UK's Joint Commission on Vaccination and Immunisation to undertake an urgent analysis to identify which adults with asthma were at an increased risk of serious COVID-19 outcomes to inform deliberations on booster COVID-19 vaccines.

**Methods:**

This national incident cohort study was done in all adults in Scotland aged 18 years and older who were included in the linked dataset of Early Pandemic Evaluation and Enhanced Surveillance of COVID-19 (EAVE II). We used data from EAVE II to investigate the risk of COVID-19 hospitalisation and the composite outcome of intensive care unit (ICU) admission or death from COVID-19 among adults with asthma. A Cox proportional hazard model was used to derive adjusted hazard ratios (HRs) and 95% CIs for the association between asthma and COVID-19 hospital admission and ICU admission or death, stratified by markers of history of an asthma attack defined by either oral corticosteroid prescription (prednisolone, prednisone, and dexamethasone) in the 2 years before March 1, 2020, or hospitalisation for asthma before March 1, 2020. Analyses were adjusted for age, sex, socioeconomic status, comorbidity, previous hospitalisation, and vaccine status.

**Findings:**

Between March 1, 2020, and July 27, 2021, 561 279 (12·7%) of 4 421 663 adults in Scotland had clinician-diagnosed-and-recorded-asthma. Among adults with asthma, 39 253 (7·0%) had confirmed SARS-CoV-2 infections, of whom 4828 (12·3%) were admitted to hospital for COVID-19 (among them, an estimated 600 [12·4%] might have been due to nosocomial infections). Adults with asthma were found to be at an increased risk of COVID-19 hospital admission (adjusted HR 1·27, 95% CI 1·23–1·32) compared with those without asthma. When using oral corticosteroid prescribing in the preceding 2 years as a marker for history of an asthma attack, the adjusted HR was 1·54 (95% CI 1·46–1·61) for those with three or more prescribed courses of oral corticosteroids, 1·37 (1·26–1·48) for those with two prescribed courses, 1·30 (1·23–1·37) for those with one prescribed course, and 1·15 (1·11–1·21) for those without any courses, compared with those aged 18 years or older without asthma. Adults with asthma were found to be at an increased risk of COVID-19 ICU admission or death compared with those without asthma (adjusted HR 1·13, 95 % CI 1·05–1·22). The adjusted HR was 1·44 (95% CI 1·31–1·58) for those with three or more prescribed courses of oral corticosteroids, 1·27 (1·09–1·48) for those with two prescribed courses, 1·04 (0·93–1·16) for those with one prescribed course, and 1·06 (0·97–1·17) for those without any course, compared with adults without asthma.

**Interpretation:**

Adults with asthma who have required two or more courses of oral corticosteroids in the previous 2 years or a hospital admission for asthma before March 1, 2020, are at increased risk of both COVID-19 hospitalisation and ICU admission or death. Patients with a recent asthma attack should be considered a priority group for booster COVID-19 vaccines.

**Funding:**

UK Research and Innovation (Medical Research Council), Research and Innovation Industrial Strategy Challenge Fund, Health Data Research UK, and Scottish Government.

## Introduction

There is emerging evidence that immunity after COVID-19 vaccination wanes, especially against the Delta variant of concern.^1^, ^2^ Many countries have been discussing booster doses to ensure that people remain protected against SARS-CoV-2 as the winter months approach. By Nov 15, 2021, more than 170 million booster doses had been administered worldwide.^3^ In the USA, booster vaccinations are available for Pfizer-BioNTech vaccine recipients who completed their initial series at least 6 months ago, including those aged 65 years and older, those aged at least 18 years who have underlying medical conditions, those who work in high-risk settings, and those who live in high-risk settings.^4^ Israel started its booster campaign aimed at those aged over 50 years and has administered over three millions third doses of Pfizer-BioNTech vaccine to date.^3^ Turkey began administering booster vaccines in July, 2021, to health-care workers and people older than 50 years.^5^ Similar initiatives are underway in other countries, including Cambodia, Thailand and Uruguay.^5^

**Research in context**

**Evidence before this study**

Understanding which adults with asthma are at an increased risk of serious COVID-19 outcomes is of critical importance in deliberations on prioritisation of booster vaccines. We searched PubMed for observational studies, with no language restrictions, using the terms “SARS-CoV-2”, “COVID-19”, “hospitalisation”, “hospital admission”, “death”, “adults”, and “asthma”, for studies published between March 1, 2020, and Sept 16, 2021. We found six studies that investigated the association between markers of asthma severity and risk of severe COVID-19 outcomes among adults. Five of these six studies showed that asthma severity, defined using different patterns of inhaled and oral corticosteroid prescribing, were associated with increased risks of serious COVID-19 outcomes. Of these five studies, only one assessed and reported an increased risk of COVID-19 hospital or intensive care unit (ICU) admission in adults with severe asthma, and the remaining four studies found that severe asthma was associated with an increased risk of COVID-19 death. The sixth, smaller study, which investigated a single hospital in England, found no association between markers of asthma severity and COVID-19 deaths, possibly because the study was underpowered. Through the peer review process, we were alerted to an additional study that assessed the risk of severe COVID-19 outcomes in adults with different asthma phenotypes. The authors found an association between markers of asthma severity and severe COVID-19 outcomes, but this was not observed in those with features suggestive of underlying type 2 inflammatory asthma.

**Added value of this study**

We report on risk factors for severe COVID-19 outcomes in adults with asthma during different waves of the pandemic, taking vaccination status into account. We found that adults with a history of an asthma attack in the preceding 24 months (defined by either two or more oral corticosteroid prescriptions or previous asthma hospitalisation) had an increased risk of COVID-19 hospital admission and the composite outcome of ICU admission or death, when compared with those with no asthma. These increased risks remained after adjusting for age, sex, socioeconomic status, comorbidity, previous non-asthma hospitalisation, and COVID-19 vaccine status. Our study has added UK evidence using nationwide population-level data and quantified the strength of associations across different waves of the pandemic, taking vaccination status into account.

**Implications of all the available evidence**

We provide national evidence that adults aged 18 years and older with a history of an asthma attack in the preceding 24 months are at an increased risk of COVID-19 hospital admission and ICU admission or death. These findings have been used by the UK Joint Commission on Vaccination and Immunisation to inform policy decisions on which adults with asthma to prioritise for COVID-19 booster vaccination.

The UK Joint Commission on Vaccination and Immunisation (JCVI) issued interim advice on COVID-19 booster vaccination on June 30, 2021, indicating that any COVID-19 booster programme should be offered to the most vulnerable people first (ie, those at the greatest risk of serious COVID-19 outcomes). The UK COVID-19 booster vaccine programme started in September, 2021, with the aim of maximising individual protection for the most vulnerable individuals and reducing the potential risk of the UK National Health Service (NHS) surge capacity being breached over the coming winter. The individuals prioritised thus far are care home residents, people aged over 40 years, frontline health and social care workers, clinically extremely vulnerable adults, and those who are immunosuppressed.^6^

There have been several reports indicating that adults with severe asthma might have an increased risk of severe COVID-19 outcomes, namely hospitalisation, intensive care unit (ICU) admission, and death.^7^, ^8^, ^9^, ^10^, ^11^, ^12^ According to the Global Initiative for Asthma (GINA), asthma severity is generally seen as a retrospective assessment of the treatment required to minimise symptoms or exacerbations, whereas asthma control relates to how a patient experiences symptoms and the risk of exacerbations.^13^ Therefore, asthma control can relate to severity, but might also be affected by adherence, inhaler technique, and exposure to triggers (eg, smoking or allergen exposure).^13^ However, the existing evidence base showing that adults with asthma are at the highest risk of serious COVID-19 outcomes is difficult to interpret, since there have been no previous studies investigating asthma risk during different waves of the pandemic and taking vaccination status into account. As a result, there is considerable uncertainty over whether adults with asthma should be offered booster vaccines against SARS-CoV-2 and, if so, who should be prioritised for booster vaccination.

In response to a request from the UK JCVI, we sought to use our national surveillance platform to investigate the risk of hospitalisation, ICU admission, and death from COVID-19 among adults with markers of history of an asthma attack in the preceding 24 months.

## Methods

### Study design

This national incident cohort study was done in all adults in Scotland aged 18 years and older who were included in the linked dataset of Early Pandemic Evaluation and Enhanced Surveillance of COVID-19 (EAVE II). EAVE II is a Scotland-wide COVID-19 surveillance platform that has been used to track and forecast the epidemiology of COVID-19, inform risk stratification assessment, and investigate vaccine effectiveness and safety.^1^, ^14^, ^15^, ^16^, ^17^, ^18^, ^19^ It comprises national health-care datasets on 5·4 million people (approximately 99% of the Scottish population) deterministically linked through the Community Health Index (CHI) number, which is a unique identifier used in all health-care contacts across NHS Scotland. We used data from EAVE II to describe the demographic profile of adults with asthma who had SARS-CoV-2 infections, COVID-19 hospital admissions, and ICU admissions or deaths. We used the composite outcome of ICU admission or death because there have been concerns about possible rationing of access to ICU beds, particularly in the early phases of the pandemic. We also undertook a national incident cohort analysis to investigate risks of hospitalisation, ICU admission, or deaths in adults with asthma, stratified by markers of history of an asthma attack. The cohort was set up on March 1, 2020 (retrospectively assigned as it was shortly before the first person was admitted to hospital due to COVID-19 in Scotland). All individuals were followed up from March 1, 2020, until the date of death or the end of follow-up (July 27, 2021), whichever came first.

Ethics approval was obtained from the National Research Ethics Service Committee, Southeast Scotland 02 (reference number 12/SS/0201). The Public Benefit and Privacy Panel Committee of Public Health Scotland approved the linkage and analysis of the de-identified datasets for this project (1920-0279).

### Data sources and procedures

The national datasets linked using CHI number were the Electronic Communication of Surveillance in Scotland (national database for all virology testing), primary care (demographics and clinical history), the Scottish Morbidity Record (which records hospitalisations), National Records of Scotland (which records mortality data), and Prescribing Information System (for prescription data). A data linkage diagram is available in the appendix (p 17).

Asthma and other risk groups of interest were measured on March 1, 2020, and defined by the QCOVID risk prediction algorithm, which consists of 30 clinical characteristics (including asthma) identified from primary care records that are known to be associated with an increased risk of serious COVID-19 outcomes in adults (appendix p 1).^20^ We excluded one risk group that had substantial missing data (ethnicity data were missing for 1 858 385 [42%] of participants; further details are given in the appendix p 3). This resulted in 28 risk groups in addition to asthma being included and analysed as potential confounders (appendix p 3).

We also assessed the risk of COVID-19 hospitalisation and ICU admission or death stratified by two markers of history of an asthma attack. First, we used previous oral corticosteroid prescribing (prednisolone, prednisone, and dexamethasone) as a marker of history of an asthma attack in the 2 years before March 1, 2020. Second, we used hospitalisation for asthma before March 1, 2020. This included all hospitalisations with a primary admission diagnosis based on International Classification of Diseases Tenth Revision codes J45 and J46 within 2 years before March 1, 2020.

Building on methods that have previously been described in detail,^17^, ^21^ we defined individuals who tested positive with real-time RT-PCR as having SARS-CoV-2 infections. We defined a COVID-19 hospital admission as being hospitalised within 14 days following a positive RT-PCR test for SARS-CoV-2, including those who tested positive while being hospitalised, or those who were hospitalised with an admission diagnosis of COVID-19 (appendix p 2). COVID-19 related deaths were all-cause deaths occurring within 28 days after a positive test for SARS-CoV-2 that were registered with National Records Scotland and included death certification, or deaths with COVID-19 on the death certificate as the cause of death.

### Statistical analysis

A Cox proportional hazard model was used to derive the hazard ratios (HR) and 95% confidence intervals (CIs) for the association between history of an asthma attack in the preceding 24 months and COVID-19 hospital admission and ICU admission or death. This model, with calendar time as the timescale, eliminates the need to model the underlying temporal trends as these are estimated as the baseline hazard. The Cox model adjusted for a penalised spline of age, sex, socioeconomic status, body-mass index (BMI), number of other risk groups of interest (ie, those identified by the QCOVID algorithm), number of non-asthma related hospitalisations within the 2-year period before March 1, 2020, and vaccine status. Socioeconomic status was determined using the Scottish Index of Multiple Deprivation (SIMD).^22^ The SIMD classification is based on deprivation quintiles: quintile 1 refers to the most deprived and quintile 5 refers to the most affluent. The SIMD was assigned according to residential postcode. BMI was categorised into less than 18·5 kg/m^2^, 18·5–24·9 kg/m^2^, 25·0–29·9 kg/m^2^, 30·0–34·9 kg/m^2^, and 35 kg/m^2^ or greater, and not recorded.^20^ Adjusted for previous hospitalisation was used as a marker of severity or health-care seeking behaviour. Vaccine status was included in the Cox model as a time-dependent variable with five statuses: no vaccination or before vaccination, within 27 days after first dose, 28 days or more after first dose, within 27 days after second dose, and 28 days or more after second dose. Individuals who had a second vaccine dose within 28 days of their first dose would not have the status 28 days after first dose and would go straight from within 27 days after first dose to within 27 days after second dose at the date of second dose. Post-hoc interaction tests were done between vaccine status and history of asthma attack in the preceding 24 months in the adjusted Cox model. If the interaction tests (likelihood ratio χ^2^ test) were significant (P<0·01), the interaction was included in the Cox model with vaccination status as an effect modifier. By contrast, if the interaction test was not significant, vaccination status was included in the model as a covariate. All analyses were carried out in adults (≥18 years). Anyone with missing SIMD or BMI data was excluded from the Cox models. We also did a post-hoc investigation to see if the magnitude of the differences between the asthma markers investigated and no asthma varied across the different waves of the pandemic (first wave: March 1, 2020, to July 31, 2020; second wave before vaccination programme started: Aug 1, 2020, to Dec 7, 2020; second wave after the vaccination programme started: Dec 8, 2020, to May 17, 2021; third wave: May 18, 2021, to end of study).

The Cox proportional hazards models used sampling weights, which were used to correct for the size of the registered general practice population being greater than the population in Scotland (in part due to individuals who had recently moved). These weights were derived by matching the age and sex numbers in the general practice data to the Scottish population data. This adjustment ensured that the denominators in the tables matched the Scottish population.

The models were fit to a dataset with all events and a random sample, without replacement, of 100 individuals per event with sample weights calculated to represent the sampling fraction and thus ensure the correct calculation of the person-years at risk for the whole population. A combined weight (sampling weights from the random sampling procedure and the weights used to correct for the size of Scottish population) was used in the statistical modelling.

A sensitivity analysis was carried out using a 1-year look back before March 1, 2020, for the markers of history of an asthma attack. We also conducted a sensitivity analysis only looking at those who tested positive for SARS-CoV-2 and measured the markers of history of an asthma attack at the date of test to see if the risk of severe COVID-19 outcome was higher in those with history of an asthma attack in the preceding 24 months following testing positive. This was to account for adults with prior oral corticosteroids prescribing or previous hospitalisation for asthma after March 1, 2020, but before their SARS-CoV-19 infections. Misdiagnosis of asthma is common in primary care, especially in older patients. We did a post-hoc stratified analysis in those with and without coexisting chronic obstructive pulmonary disease (COPD) and investigated the association between history of an asthma attack in the preceding 24 months and COVID-19 hospitalisation within each stratum. We also did a post-hoc subgroup analysis only including those younger than 50 years to minimise the risk of confounding by COPD and a subgroup analysis reporting on separate ICU admission and mortality outcomes.

We followed the strengthening the STROBE checklist^23^ to guide transparent reporting of this cohort study (appendix pp 4–5). Analyses were done in R version 3.6.1.

### Role of the funding source

The funder of the study had no role in study design, data collection, data analysis, data interpretation, or the writing of the report.

## Results

4 421 663 adults in the EAVE II linked dataset who were aged 18 years or older on March 1, 2020, were included in the analysis. 561 279 (12·7%) adults had clinician-diagnosed-and-recorded asthma. Among adults with asthma, 39 253 (7·0%) had confirmed SARS-CoV-2 infections, of whom 4828 (12·3%) were admitted to hospital for COVID-19 (among them, an estimated 600 [12·4%] might have been due to nosocomial infections). There were 1600 (4·1%) ICU admissions and 1206 (3·1%) deaths, of which 1186 (98·3%) had COVID-19 recorded as the cause of death on the death certificate. The baseline characteristics for adults with asthma stratified by markers of history of an asthma attack are available in the appendix (pp 6–7).

The numbers of adults being tested for SARS-CoV-2, testing positive, and being admitted to hospital with COVID-19 per 100 000 people were higher in adults with asthma (irrespective of history of an asthma attack) compared with those without asthma (table 1). The number of COVID-19 ICU admissions or deaths in adults without a recent asthma attack was similar to those without asthma, but higher in those with a history of an asthma attack in the preceding 2 years. Adults with a history of an asthma attack in the preceding 2 years (defined by either previous oral corticosteroid prescription or asthma hospitalisation) had higher rates of COVID-19 hospitalisation and ICU admission or death compared with those with better-controlled asthma. The absolute rates of COVID-19 hospitalisation in adults with three or more, two, one, or zero prescribed courses of oral corticosteroids were 2375, 1600, 1274, and 642 per 100 000 people, respectively. The absolute rate of COVID-19 hospitalisation was 3290 per 100 000 people in adults with previous hospitalisation for asthma and 835 per 100 000 people in adults without previous hospitalisation for asthma.

**Table 1:** Number and rate (per 100 000 people) of being tested, testing positive, COVID-19 hospitalisation, ICU admission, and deaths in adults with asthma, stratified by markers of history of an asthma attack

	**Overall number**	**Number of patients tested**	**Number testing positive**	**Number of patients hospitalised with COVID-19**	Number of ICU admissions or deaths*	**Number of ICU admissions**	**Number of deaths**
**Asthma**							
No	3 860 383	1 500 203 (38·9%)	225 052 (5·8%)	23 861 (0·6%)	10 693 (0·3%)	2463 (0·1%)	8913 (0·2%)
Yes	561 279	271 374 (48·3%)	39 253 (7·0%)	4828 (0·9%)	1600 (0·3%)	516 (0·1%)	1206 (0·2%)
**History of an asthma attack (OCS prescription**†**)**							
No asthma	3 680 316	1 414 860 (38·4%)	215 345 (5·9%)	20 678 (0·6%)	9193 (0·2%)	2198 (0·1%)	7561 (0·2%)
Asthma with no courses of OCS	440 388	204 035 (46·3%)	30 944 (7·0%)	2827 (0·6%)	955 (0·2%)	297 (0·1%)	732 (0·2%)
Asthma with one course of OCS	142 583	71 258 (50·0%)	9154 (6·4%)	1817 (1·3%)	681 (0·5%)	171 (0·1%)	568 (0·4%)
Asthma with two courses of OCS	50 880	26 240 (51·6%)	3124 (6·1%)	814 (1·6%)	330 (0·6%)	88 (0·2%)	268 (0·5%)
Asthma with three or more courses of OCS	107 496	55 184 (51·3%)	5738 (5·3%)	2553 (2·4%)	1134 (1·1%)	225 (0·2%)	990 (0·9%)
**History of an asthma attack (previous hospitalisation**‡**)**							
No asthma§	3 859 720	1 499 858 (38·9%)	225 009 (5·8%)	23 845 (0·6%)	10 689 (0·3%)	2463 (0·1%)	8909 (0·2%)
Asthma without previous hospitalisation	555 833	267 681 (48·2%)	38 790 (7·0%)	4643 (0·8%)	1557 (0·3%)	498 (0·1%)	1178 (0·2%)
Asthma with previous hospitalisation	6110	4038 (66·1%)	506 (8·2%)	201 (3·3%)	47 (0·3%)	18 (0·3%)	32 (0·5%)
**History of an asthma attack (previous hospitalisation**‡**or OCS prescription**†**)**							
No asthma§	3 680 068	1 414 738 (38·4%)	215 332 (5·9%)	20 675 (0·6%)	9192 (0·2%)	2198 (0·1%)	7560 (0·2%)
Asthma with 0–1 course(s) of OCS and no previous hospitalisation	580 684	273 907 (47·2%)	39 915 (6·9%)	4590 (0·8%)	1625 (0·3%)	465 (0·1%)	1292 (0·2%)
Asthma with ≥2 courses of OCS or previous hospitalisation	160 910	82 932 (51·5%)	9058 (5·6%)	3424 (2·1%)	1476 (0·9%)	316 (0·2%)	1267 (0·8%)

Data are n or n (%). Denominators of the percentages are those listed in the overall number column. ICU=intensive care unit. OCS=oral corticosteroid.

* ICU deaths referred to those who had COVID-19-related ICU admissions or COVID-19-related death with or without previous ICU admissions.

† OCS prescriptions for prednisolone, prednisone, and dexamethasone in the 2-year period before March 1, 2020.

‡ Hospitalisation for asthma within 2-year period before March 1, 2020.

§ The no asthma group under variable asthma was derived using only the general practitioner-recorded diagnosis whereas the no asthma group under the variable history of an asthma attack (OCS prescription) was derived using both general practitioner diagnosis and prescribing records; therefore, patients who never had asthma recorded in their general practice records, but had OCS prescriptions were not included in the second no asthma group, but were included in the first asthma group (using only general practitioner records), which explains the smaller group size.

Adults with asthma were found to be at an increased risk of COVID-19 hospital admission (adjusted HR 1·27, 95% CI 1·23–1·32) compared with those without asthma. When using oral corticosteroid prescribing in the preceding 2 years as a marker for history of an asthma attack, the adjusted HR was 1·54 (95% CI 1·46–1·61) for those with three or more prescribed courses of oral corticosteroids, 1·37 (1·26–1·48) for those with two prescribed courses, 1·30 (1·23–1·37) for those with one prescribed course, and 1·15 (1·11–1·21) for those without any courses, compared with those aged 18 years or older without asthma (table 2). 1 858 385 (42%) of the cohort had missing ethnicity so this variable was not adjusted for in the Cox model. 43 467 (1%) of the cohort had missing BMI and 42 132 (1%) of the cohort had missing SIMD and were excluded from the adjusted Cox model. Vaccination was slightly less effective in reducing COVID-19 hospitalisation in those with a history of an asthma attack as measured by oral corticosteroid use in the preceding 2 years compared with those without asthma (P_interaction_=0·0020). For those without asthma, the adjusted HR of at 28 days or more after the second dose of vaccine versus unvaccinated individuals was 0·15 (95% CI 0·14–0·17), whereas for those with three or more courses of oral corticosteroids the adjusted HR was 0·23 (0·19–0·28; figure; appendix p 8). The difference in COVID-19 hospitalisations between those with a history of asthma attack as measured by oral corticosteroid use and those without asthma was larger in the third wave of the COVID-19 pandemic (adjusted HR for three or more courses of oral corticosteroids *vs* no asthma 2·05, 95% CI 1·75–2·39 compared with 1·56, 1·40–1·74 in the first wave of the COVID-19 pandemic; appendix p 9).

**Table 2:** HRs for COVID-19 hospitalisation, ICU admissions, or deaths for those with different markers of history of an asthma attack and those with no asthma in adults

	**COVID-19 hospitalisation**		COVID-19 ICU admissions or deaths*	
	Number of events	Adjusted HR (95% CI)	Number of events	Adjusted HR (95% CI)
**Previous prescribed OCS as a marker of history of an asthma attack**				
No asthma	20 678	1 (ref)	9193	1 (ref)
Asthma with no course of OCS	2827	1·15 (1·11–1·21)	955	1·06 (0·97–1·17)
Asthma with one course of OCS	1817	1·30 (1·23–1·37)	681	1·04 (0·93–1·16)
Asthma with two courses of OCS	814	1·37 (1·26–1·48)	330	1·27 (1·09–1·48)
Asthma with three or more courses of OCS	2553	1·54 (1·46–1·61)	1134	1·44 (1·31–1·58)
**Previous hospitalisation for asthma as a marker of history of an asthma attack**				
No asthma	23 845	1 (ref)	10 689	1 (ref)
Asthma without previous hospitalisation	4643	1·24 (1·20–1·29)	1557	1·11 (1·03–1·19)
Asthma with previous hospitalisation	201	3·01 (2·59–3·49)	47	2·24 (1·56–3·20)

HRs were derived using Cox proportional hazard models adjusted for age, sex, socioeconomic status, body-mass index, number of risk groups of interest, number of non-asthma-related hospitalisations within the 2-year period before March 1, 2020, and vaccine status. HR=hazard ratio. ICU=intensive care unit. OCS=oral corticosteroid.

* ICU admissions or deaths referred to those who had COVID-19-related ICU admissions or COVID-19-related death with or without previous ICU admissions.

**Figure:**
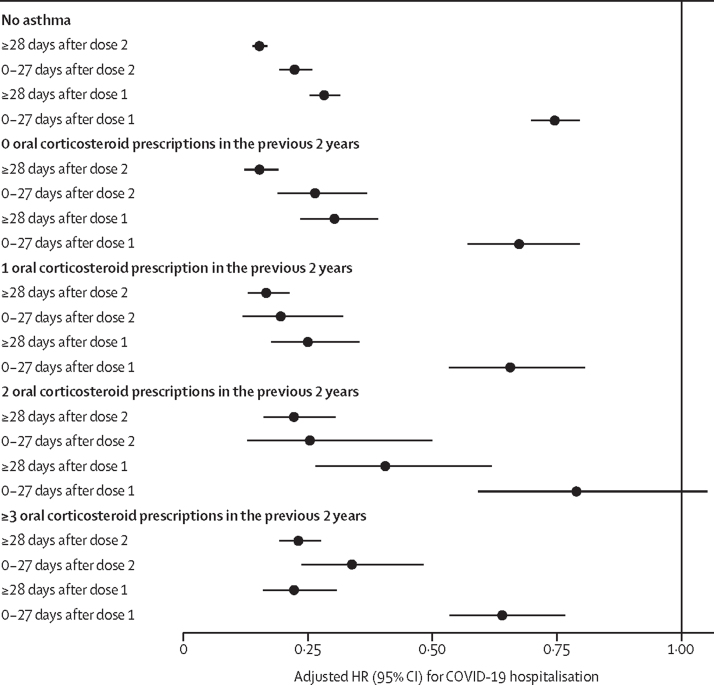
Vaccine protection against COVID-19 hospitalisation stratified by markers of an asthma attack, defined by previous oral corticosteroid course in the 2 years before March 1, 2020 HRs were derived using Cox proportional hazard models adjusted for age, sex, socioeconomic status, body-mass index, number of risk groups of interest, and number of non-asthma-related hospitalisations within the 2-year period before March 1, 2020. The reference level for the adjusted HR is the unvaccinated group. HR=hazard ratio.

When using previous hospitalisation for asthma as the marker of history of an asthma attack, the adjusted HR was 3·01 (95% CI 2·59–3·49) for those with hospitalisation for asthma in the previous 2 years and 1·24 (1·20–1·29) for those without hospitalisation for asthma in the previous 2 years compared with those aged 18 years and older without asthma (table 2).

Adults with asthma were found to be at an increased risk of COVID-19 ICU admission or death compared with those without asthma (adjusted HR 1·13, 95 % CI 1·05–1·22). The adjusted HR was 1·44 (95% CI 1·31–1·58) for those with three or more prescribed courses of oral corticosteroids, 1·27 (1·09–1·48) for those with two prescribed courses, 1·04 (0·93–1·16) for those with one prescribed course, and 1·06 (0·97–1·17) for those without any course, compared with adults without asthma (table 2). The adjusted HR was 2·24 (95% CI 1·56–3·20) for those with previous hospitalisation for asthma and 1·11 (1·03–1·19) for those with no previous hospitalisation for asthma compared with those aged 18 years and older without asthma (table 2). Separate analyses for ICU admission and death are shown in the appendix (p 10).

The sensitivity analyses using 1-year retrospective data up to March 1, 2020, for the two markers of history of an asthma attack also yielded similar results (appendix p 11). Sensitivity analyses focusing on those who tested positive for COVID-19 and measuring the markers of history of an asthma attack at the date of test showed similar results (appendix p 12). For the group of people without COPD, asthma had a stronger effect on COVID-19 hospitalisation compared with the group of people with COPD (appendix p 13). Similarly, for people younger than 50 years, asthma had a stronger effect on severe COVID-19 outcomes (ICU admission or death) compared with the general population (appendix p 14). The univariable analysis for all the risk factors, including COPD, is available in the appendix (p 15).

## Discussion

We found that adults with a prescription of two or more courses of oral corticosteroids or asthma hospitalisation in the preceding 2 years are at an increased risk of both COVID-19 hospitalisation and ICU admission or death compared with those without asthma. This would translate into 160 910 adults with asthma aged 18 years or older who have received two or more courses of oral corticosteroids or previous hospitalisation for asthma in Scotland during the study period who might be prioritised for COVID-19 vaccines, which when scaled-up to the UK would equate to around 1 930 920 adults (assuming the same prevalence of severe asthma in the other UK nations).^24^ If we restricted our analysis to only those who received two or more courses of oral corticosteroids in the preceding 2 years, this would translate into around 158 000 adults in Scotland, which is similar to the number (around 160 000) if we used both markers of history of an asthma attack (previous asthma hospitalisation or two or more courses of oral corticosteroids in the preceding 2 years). There might be some protection against severe COVID-19 in those who did not have a recent asthma attack, but our findings were not significant.

Our study has several strengths. We developed a national linked dataset and have created a platform that allowed rapid access to and analysis of data from routinely collected national electronic health record data. Therefore, this study is less susceptible to recall or misclassification bias than are studies that rely on primary data collection. The use of a large population aided study power, facilitating estimation of HRs in different markers of history of an asthma attack and different outcomes. The study is likely to have excellent generalisability across the UK and potentially across other countries with similar demographics and health systems. Finally, we have been able to show that the associations found were similar across different phases of the pandemic (and hence across variants in circulation).

Our study has several limitations. There were relatively small absolute numbers of people with previous asthma hospitalisations, so these data should be interpreted with care. However, our results were broadly consistent across different measures of previous asthma attack. We were unable to assess the association between asthma severity or control, as defined by GINA, and COVID-19-related risks. We included 28 risk groups that were defined by the QCOVID prediction algorithm,^21^ but we might have missed some important risk groups. Adults with a history of an asthma attack in the preceding 2 years had an increased rate of being tested compared with those with no recent asthma or without asthma. This might be because they could be more likely to be admitted to hospital and therefore more likely to have routine SARS-CoV-2 testing and screening in hospital than those with no recent asthma attack or without asthma. This could partly explain why there was little difference between the waves in effect estimates for asthma. There might also have been different health-care seeking behaviours among adults with a history of asthma attack, which might have resulted in increased chances of being tested for SARS-CoV-2. Although our Cox models were adjusted for potential confounders, unmeasured confounders could still have influenced our estimates. Our analysis did not include some potentially important confounders (such as ethnicity) because of the lack of reliable recording of this variable within Scottish electronic health records, with the consequence that residual confounding remains a possibility. Prescribing of oral corticosteroids was in people with a history of asthma, so our assumption is that these steroids were given for an asthma attack. However, we cannot be sure that this was the case. The indication for treatment and length of the prescription would have been helpful in this respect, but these data were unfortunately unavailable within our dataset.

Similar findings have been reported elsewhere.^7^, ^8^, ^9^, ^10^, ^11^, ^12^ Specifically, five studies found an increased risk of COVID-19 death and two studies found an increased risk of COVID-19 hospital or ICU admission in adults with severe asthma.^7^, ^8^, ^9^, ^10^, ^11^, ^12^ No association was observed between severe asthma and COVID-19 deaths in one study, which could have been because of the small study sample size, which only included data from a single English hospital.^25^ Particularly high risks of COVID-19 hospital admission, ICU admission, and death have been reported in patients with asthma using high doses of inhaled corticosteroids and those with recent oral corticosteroid use for asthma (in the past 1–2 years).^7^, ^8^, ^9^, ^12^ Our study has contributed to UK evidence using nationwide population-level data and quantified the strength of associations between history of an asthma attack in the preceding 2 years and markers of severe COVID-19 outcomes across different waves of the COVID-19 pandemic, accounting for vaccination status. Our analysis shows that these findings remained robust, regardless of the wave of the pandemic, public behaviour, changes in clinical management, and vaccination policy.

Building on this work, it is important to characterise in more detail the markers of history of an asthma attack for severe COVID-19 outcomes in adults and to investigate underlying mechanisms that predispose such adults to these increased risks. This analysis underscores the importance of maintaining good asthma control and careful monitoring of adults with history of an asthma attack if they develop SARS-CoV-2 infection. The finding that two vaccination doses were effective in reducing the risk of serious COVID-19 outcomes in those with a previous recent asthma attack, but less so than in those without asthma, underscores the need for additional vaccine doses in this subsection of the population with asthma. With booster vaccines being administered or planned internationally and nationally, together with other public health surveillance data, policy makers will be able to use data from our study to inform decisions on booster vaccination priorities among adults with asthma.

In conclusion, we provide national evidence that adults with two or more courses of oral corticosteroids or asthma admission in the preceding 2 years were associated with an increased risk of COVID-19 hospital admission and ICU admission or death in Scotland. The findings from this linkage of multiple data sources have helped inform UK policy deliberations on vaccine boosters for adults with asthma.

## Data sharing

A data dictionary covering the data sources used in this study can be found at https://github.com/EAVE-II/EAVE-II-data-dictionary. The read codes for the QCOVID risk groups were sent directly from the University of Oxford-based QCOVID team to the Scottish Government and from there to Health Education Scotland, who then transferred the codes to Albasoft for extraction of risk groups from the general practitioner data. Because of this procedure, which was required by the governance permissions, we did not have access to the individual read codes. All code developed for this analysis is available online at https://github.com/EAVE-II/Covid-asthma-adults. We will also deposit the meta-data in Health Data Research Innovation Gateway. The data used in this study are sensitive because of individual patient-level data and will not be made publicly available.

## Declaration of interests

AS and CR are members of the Scottish Government's Chief Medical Officer COVID-19 Advisory Group. AS and CR are members of the New and Emerging Respiratory Virus Threats Advisory Group risk stratification subgroup. CR is a member of the Scientific Pandemic Influenza Group on Modelling. AS is a member of AstraZeneca's Thrombotic Thrombocytopenic Advisory Group and the Scottish Government's Standing Committee on Pandemics. AS is Director of the Asthma UK Centre for Applied Research. All aforementioned roles are unremunerated. All other authors report no competing interests.
