# Supplementary material for: Risk of serious COVID-19 outcomes among adults with asthma in Scotland: a national incident cohort study
Source: Lancet Respir Med. 2022 Apr;10(4):347–54. doi: 10.1016/S2213-2600(21)00543-9 (PMC8758152; doi:10.1016/S2213-2600(21)00543-9)
Supplement: Supplementary appendix [file mmc1.pdf]

THE LANCET
Respiratory Medicine

Supplementary appendix

This appendix formed part of the original submission and has been peer reviewed. We post it as supplied by the authors.

Supplement to: Shi T, Pan J, Vasileiou E, Robertson C, Sheikh A, on behalf of Public Health Scotland and the EAVE II Collaborators. Risk of serious COVID-19 outcomes among adults with asthma in Scotland: a national incident cohort study. *Lancet Respir Med* 2022; published online Jan 13. https://doi.org/10.1016/S2213-2600(21)00543-9.

Supplementary material

Box 1: Predictor variables in the QCOVID algorithm

- accommodation (homeless, care home, neither)
- asthma
- atrial fibrillation
- blood cancer
- body mass index (BMI)
- cerebral palsy
- chronic kidney disease
- cirrhosis of liver
- congenital heart disease
- congestive cardiac failure
- chronic obstructive pulmonary disease (COPD)
- coronary heart disease
- dementia
- diabetes 1
- diabetes 2
- epilepsy
- ethnicity
- learning disability
- osteoporotic fracture
- Parkinson's disease
- peripheral vascular disease
- pulmonary hypertension or pulmonary fibrosis
- rare neurological conditions
- rare pulmonary diseases
- respiratory cancer
- rheumatoid arthritis or systemic lupus erythematosus
- severe mental illness
- sickle cell disease
- stroke
- venous thromboembolism

Table S1: ICD-10 codes for COVID-19 illness

Code	Description
U07.1	COVID-19, virus identified
U07.2	COVID-19, virus not identified
Source: https://www.who.int/classifications/icd/COVID-19-coding-icd10.pdf	

ICD-10: International Classification of Diseases 10.

Table S2: List of 28 other risk groups of interest included in this study

**Risk group of interest***	**Coding**
Accommodation	Homeless/care home/neither
Atrial fibrillation	Yes/no
Blood cancer	Yes/no
Body mass index	<18.5, 18.5-24.99, 25-29.99, 30-34.99, ≥35, and not recorded
Cerebral palsy	Yes/no
Chronic kidney disease	Yes/no
Cirrhosis of liver	Yes/no
Congenital heart disease	Yes/no
Congestive cardiac failure	Yes/no
Chronic obstructive pulmonary disease	Yes/no
Coronary heart disease	Yes/no
Dementia	Yes/no
Diabetes type 1	Yes/no
Diabetes type 2	Yes/no
Epilepsy	Yes/no
Learning disability	Yes without Down’s syndrome/yes with Down’s syndrome/no
Osteoporotic fracture	Yes/no
Parkinson’s disease	Yes/no
Peripheral vascular disease	Yes/no
Pulmonary hypertension or pulmonary fibrosis	Yes/no
Rare neurological conditions	Yes/no
Rare pulmonary diseases	Yes/no
Respiratory cancer	Yes/no
rheumatoid arthritis or systemic lupus erythematosus	Yes/no
Severe mental illness	Yes/no
Sickle cell disease	Yes/no
Stroke	Yes/no
Venous thromboembolism	Yes/no

*Ethnicity data were missing on 42% of participants therefore not included.

Table S3: Reporting STROBE checklist for cohort studies

	Item No	Recommendation	Location
**Title and abstract**	1	(*a*) Indicate the study's design with a commonly used term in the title or the abstract	Page 2
		(*b*) Provide in the abstract an informative and balanced summary of what was done and what was found	Page 2
**Introduction**			
Background/rationale	2	Explain the scientific background and rationale for the investigation being reported	Page 4
Objectives	3	State specific objectives, including any prespecified hypotheses	Page 4
**Methods**			
Study design	4	Present key elements of study design early in the paper	Page 4
Setting	5	Describe the setting, locations, and relevant dates, including periods of recruitment, exposure, follow-up, and data collection	Page 4
Participants	6	(*a*) Give the eligibility criteria, and the sources and methods of selection of participants. Describe methods of follow-up	Page 4
		(*b*) For matched studies, give matching criteria and number of exposed and unexposed	NA
Variables	7	Clearly define all outcomes, exposures, predictors, potential confounders, and effect modifiers. Give diagnostic criteria, if applicable	Page 4-5
Data sources/ measurement	8*	For each variable of interest, give sources of data and details of methods of assessment (measurement). Describe comparability of assessment methods if there is more than one group	Page 5
Bias	9	Describe any efforts to address potential sources of bias	Page 5
Study size	10	Explain how the study size was arrived at	Page 5
Quantitative variables	11	Explain how quantitative variables were handled in the analyses. If applicable, describe which groupings were chosen and why	Page 5
Statistical methods	12	(*a*) Describe all statistical methods, including those used to control for confounding	Pages 5-6
		(*b*) Describe any methods used to examine subgroups and interactions	Page 6
		(*c*) Explain how missing data were addressed	Page 5
		(*d*) If applicable, explain how loss to follow-up was addressed	NA
		(*e*) Describe any sensitivity analyses	NA
**Results**			
Participants	13*	(a) Report numbers of individuals at each stage of study—eg numbers potentially eligible, examined for eligibility, confirmed eligible, included in the study, completing follow-up, and analysed	Page 7
		(b) Give reasons for non-participation at each stage	NA
		(c) Consider use of a flow diagram	Page 5 Figure S1
Descriptive data	14*	(a) Give characteristics of study participants (eg demographic, clinical, social) and information on exposures and potential confounders	Page 7
		(b) Indicate number of participants with missing data for each variable of interest	Page 5, Table S2
		(c) Summarise follow-up time (eg, average and total amount)	Page 5

Outcome data	15*	Report numbers of outcome events or summary measures over time	Page 7
Main results	16	(*a*) Give unadjusted estimates and, if applicable, confounder-adjusted estimates and their precision (eg, 95% confidence interval). Make clear which confounders were adjusted for and why they were included	Pages 7-8 Table S12
		(*b*) Report category boundaries when continuous variables were categorized	NA
		(*c*) If relevant, consider translating estimates of relative risk into absolute risk for a meaningful time period	NA
Other analyses	17	Report other analyses done—eg analyses of subgroups and interactions, and sensitivity analyses	Page 8
**Discussion**			
Key results	18	Summarise key results with reference to study objectives	Page 8
Limitations	19	Discuss limitations of the study, taking into account sources of potential bias or imprecision. Discuss both direction and magnitude of any potential bias	Page 9
Interpretation	20	Give a cautious overall interpretation of results considering objectives, limitations, multiplicity of analyses, results from similar studies, and other relevant evidence	Page 9
Generalisability	21	Discuss the generalisability (external validity) of the study results	Page 9
**Other information**			
Funding	22	Give the source of funding and the role of the funders for the present study and, if applicable, for the original study on which the present article is based	Page 10

*Give information separately for exposed and unexposed groups. STROBE: Strengthening the Reporting of Observational studies in Epidemiology. NA: not applicable.

Table S4: Baseline characteristics of the included population

Variable	Level	Overall number	With asthma	Asthma with no prior hospitalisation*	Asthma with prior hospitalisation*	Asthma with 0 OCS^	Asthma with 1 OCS^	Asthma with 2 OCS^	Asthma with ≥3 OCS^
Total	Total	4421663 (100)	561279 (100)	555833 (100)	6110 (100)	440388 (100)	142583 (100)	50880 (100)	107496 (100)
Age	18-64 years old	3385418 (76.6)	452112 (80.6)	447923 (80.6)	4641 (76)	368224 (83.6)	93443 (65.5)	30648 (60.2)	49186 (45.8)
Age	65-79 years old	766350 (17.3)	83994 (15)	83069 (14.9)	1042 (17.1)	55578 (12.6)	35802 (25.1)	14793 (29.1)	40731 (37.9)
Age	≥80 years old	269895 (6.1)	25173 (4.5)	24841 (4.5)	426 (7)	16586 (3.8)	13339 (9.4)	5439 (10.7)	17579 (16.4)
Prior hospitalisation§	0	3516527 (79.5)	427119 (76.1)	424321 (76.3)	3078 (50.4)	351579 (79.8)	90435 (63.4)	28968 (56.9)	47835 (44.5)
Prior hospitalisation	1	533418 (12.1)	78336 (14)	77167 (13.9)	1301 (21.3)	55212 (12.5)	26378 (18.5)	10059 (19.8)	23200 (21.6)
Prior hospitalisation	2	187268 (4.2)	28377 (5.1)	27790 (5)	658 (10.8)	18171 (4.1)	11333 (7.9)	5013 (9.9)	13264 (12.3)
Prior hospitalisation	≥3	184450 (4.2)	27447 (4.9)	26554 (4.8)	1073 (17.6)	15426 (3.5)	14437 (10.1)	6840 (13.4)	23197 (21.6)
Sex	Female	2288128 (51.7)	304213 (54.2)	300283 (54)	4371 (71.5)	224167 (50.9)	87428 (61.3)	32133 (63.2)	67594 (62.9)
Sex	Male	2133535 (48.3)	257066 (45.8)	255550 (46)	1738 (28.4)	216221 (49.1)	55155 (38.7)	18747 (36.8)	39902 (37.1)
SIMD#	1 - High	871447 (19.7)	123595 (22)	121928 (21.9)	1856 (30.4)	92083 (20.9)	35346 (24.8)	13275 (26.1)	26760 (24.9)
SIMD	2	869700 (19.7)	116795 (20.8)	115476 (20.8)	1502 (24.6)	89666 (20.4)	31531 (22.1)	11624 (22.8)	24285 (22.6)
SIMD	3	875083 (19.8)	108219 (19.3)	107229 (19.3)	1130 (18.5)	85011 (19.3)	28119 (19.7)	9911 (19.5)	21530 (20)
SIMD	4	876772 (19.8)	107601 (19.2)	106788 (19.2)	897 (14.7)	86661 (19.7)	25658 (18)	8756 (17.2)	19083 (17.8)
SIMD	5 - Low	886527 (20)	101481 (18.1)	100852 (18.1)	686 (11.2)	83999 (19.1)	21042 (14.8)	7011 (13.8)	15199 (14.1)

SIMD	NA	42132 (1)	3589 (0.6)	3560 (0.6)	39 (0.6)	2966 (0.7)	886 (0.6)	302 (0.6)	639 (0.6)
BMI (kg/m2)	<18.5	49520 (1.1)	7039 (1.3)	6961 (1.3)	84 (1.4)	5218 (1.2)	2036 (1.4)	902 (1.8)	2519 (2.3)
BMI	18.5-24.99	550329 (12.4)	77429 (13.8)	76781 (13.8)	698 (11.4)	60401 (13.7)	18847 (13.2)	7099 (14)	17005 (15.8)
BMI	25-29.99	3055117 (69.1)	336209 (59.9)	333744 (60)	2947 (48.2)	278212 (63.2)	79167 (55.5)	26351 (51.8)	53171 (49.5)
BMI	30-34.99	430478 (9.7)	74591 (13.3)	73599 (13.2)	1043 (17.1)	52750 (12)	21725 (15.2)	8158 (16)	16728 (15.6)
BMI	≥35	292751 (6.6)	61009 (10.9)	59829 (10.8)	1237 (20.2)	40848 (9.3)	17855 (12.5)	7025 (13.8)	13300 (12.4)
BMI	NA	43467 (1)	5003 (0.9)	4919 (0.9)	101 (1.7)	2958 (0.7)	2953 (2.1)	1344 (2.6)	4773 (4.4)
Number of risk groups	0	2971660 (67.2)	330585 (58.9)	328349 (59.1)	2715 (44.4)	279187 (63.4)	69403 (48.7)	21403 (42.1)	34320 (31.9)
Number of risk groups	1	914063 (20.7)	141512 (25.2)	139896 (25.2)	1710 (28)	106660 (24.2)	37616 (26.4)	13704 (26.9)	27903 (26)
Number of risk groups	2	311003 (7)	50868 (9.1)	50020 (9)	902 (14.8)	33063 (7.5)	18453 (12.9)	7902 (15.5)	20518 (19.1)
Number of risk groups	3	129051 (2.9)	21542 (3.8)	21138 (3.8)	419 (6.9)	12600 (2.9)	9145 (6.4)	4124 (8.1)	12341 (11.5)
Number of risk groups	4	57234 (1.3)	9727 (1.7)	9522 (1.7)	213 (3.5)	5286 (1.2)	4530 (3.2)	2074 (4.1)	6875 (6.4)
Number of risk groups	≥5	38652 (0.9)	7045 (1.3)	6909 (1.2)	151 (2.5)	3592 (0.8)	3438 (2.4)	1673 (3.3)	5539 (5.2)

Data are n (%). * Hospitalisation for asthma within two-year period prior to March 1, 2020. ^ Oral corticosteroids prescriptions for prednisolone, prednisone and dexamethasone in two-year period prior to March 1, 2020. § Number of non-asthma hospitalisations within two-year period prior to March 1, 2020. # 1 indicates most deprived, 5 indicates least deprived. OCS: oral corticosteroid. SIMD: Scottish Index of Multiple Deprivation. BMI: body mass index. NA: not available.

Table S5: Hazard ratio for COVID-19 hospitalisation comparing those with different vaccine status and those with no vaccine stratified by markers of an asthma attack defined by the OCS use in the two years prior to March 1, 2020

Vaccine status	OCS courses as marker of history of an asthma attack	Number of event	aHR (95% CI)
Unvaccinated	no asthma	17950	1
0-27 days post 1st dose	no asthma	1262	0.75 (0.7-0.8)
28+ days post 1st dose	no asthma	439	0.28 (0.25-0.31)
0-27 days post 2nd dose	no asthma	222	0.22 (0.19-0.26)
28+ days post 2nd dose	no asthma	665	0.15 (0.14-0.17)
Unvaccinated	0	2448	1
0-27 days post 1st dose	0	166	0.67 (0.57-0.8)
28+ days post 1st dose	0	67	0.3 (0.24-0.39)
0-27 days post 2nd dose	0	38	0.26 (0.19-0.37)
28+ days post 2nd dose	0	94	0.15 (0.12-0.19)
Unvaccinated	1	1577	1
0-27 days post 1st dose	1	107	0.66 (0.53-0.81)
28+ days post 1st dose	1	35	0.25 (0.18-0.35)
0-27 days post 2nd dose	1	17	0.2 (0.12-0.32)
28+ days post 2nd dose	1	74	0.17 (0.13-0.21)
Unvaccinated	2	680	1
0-27 days post 1st dose	2	55	0.79 (0.59-1.05)
28+ days post 1st dose	2	24	0.41 (0.27-0.62)
0-27 days post 2nd dose	2	9	0.25 (0.13-0.5)
28+ days post 2nd dose	2	44	0.22 (0.16-0.31)
Unvaccinated	3+	2165	1
0-27 days post 1st dose	3+	140	0.64 (0.54-0.77)
28+ days post 1st dose	3+	40	0.22 (0.16-0.31)
0-27 days post 2nd dose	3+	34	0.34 (0.24-0.48)
28+ days post 2nd dose	3+	152	0.23 (0.19-0.28)

aHR: adjusted hazard ratio. CI: confidence interval. OCS: oral corticosteroid. Hazard ratios were derived using cox proportional hazard model adjusting for age, sex, socioeconomic status, body mass index, number of risk groups of interest, number of non-asthma related hospitalisations within the two-year period prior to March 1, 2020. 42% of the cohort have missing ethnicity so this variable is not adjusted for in the Cox model and 1% of the cohort have missing SIMD or BMI and this 1% were excluded in the adjusted Cox model. * Two-year look back from March 1, 2020. Interaction test p value 0.002.

Table S6: Hazard ratio for COVID-19 hospitalisation and COVID-19 ICU admissions/ deaths comparing those with markers of an asthma attack defined by the OCS use in the two years prior to March 1, 2020 stratified by different time periods during pandemic

	COVID-19 hospitalisation		COVID-19 ICU/deaths	
Asthma severity marker defined by prior OCS courses	Number of events	aHR (95% CI)	Number of events	aHR (95% CI)
March 1, 2020 to July 31, 2020				
No asthma	4883	1	3518	1
Asthma with 0 course of OCS	658	1.21 (1.1-1.33)	308	1.24 (0.92-1.67)
Asthma with 1 course of OCS	451	1.27 (1.13-1.44)	243	0.89 (0.53-1.48)
Asthma with 2 courses of OCS	191	1.3 (1.08-1.55)	102	1.94 (1.13-3.34)
Asthma with ≥3 courses of OCS	686	1.56 (1.4-1.74)	398	1.9 (1.27-2.84)
August 1, 2020 to December 7, 2020				
No asthma	5331	1	1758	1
Asthma with 0 course of OCS	698	1.1 (1.01-1.2)	190	0.92 (0.68-1.24)
Asthma with 1 course of OCS	479	1.27 (1.14-1.41)	132	1.31 (0.91-1.89)
Asthma with 2 courses of OCS	225	1.3 (1.11-1.52)	76	2.06 (1.3-3.24)
Asthma with ≥3 courses of OCS	666	1.4 (1.27-1.54)	224	1.97 (1.42-2.75)
December 8, 2020 to May 18, 2021				
No asthma	8328	1	3572	1
Asthma with 0 course of OCS	1142	1.14 (1.07-1.21)	412	1.27 (1.04-1.54)
Asthma with 1 course of OCS	734	1.3 (1.21-1.41)	284	1.05 (0.95-1.17)
Asthma with 2 courses of OCS	313	1.33 (1.19-1.49)	141	1.03 (0.91-1.16)
Asthma with ≥3 courses of OCS	973	1.54 (1.44-1.65)	460	1.2 (1-1.42)
May 19, 2021 to 27 July, 2021				
No asthma	1996	1	345	1
Asthma with 0 course of OCS	315	1.15 (1.02-1.3)	45	1.05 (0.77-1.44)
Asthma with 1 course of OCS	146	1.39 (1.17-1.65)	22	1.06 (0.69-1.65)
Asthma with 2 courses of OCS	83	1.99 (1.59-2.49)	11	1.29 (0.71-2.36)
Asthma with ≥3 courses of OCS	206	2.05 (1.75-2.39)	52	2.24 (1.63-3.08)

aHR: adjusted hazard ratio. CI: confidence interval. ICU: intensive care unit. OCS: oral corticosteroid. Hazard ratios were derived using cox proportional hazard model adjusting for age, sex, socioeconomic status, body mass index, number of risk groups of interest, number of non-asthma related hospitalisations within the two-year period prior to March 1, 2020 and vaccine status. ICU/deaths referred to those who had COVID-19 related ICU admissions or COVID-19 related death with or without previous ICU admissions. 42% of the cohort had missing ethnicity so this variable was not adjusted for in the Cox model. 1% of the cohort had missing SIMD or BMI and this 1% were excluded in the adjusted Cox model. ICU/deaths include those had COVID-19 related ICU admissions or COVID-19 related death with or without previous ICU admissions.

Table S7: Hazard ratio for COVID-19 hospitalisation, ICU admission and deaths comparing those with different markers of history of an asthma attack (defined in the two years prior to March 1, 2020) and those with no asthma in adults

	COVID-19 hospitalisation		COVID-19 ICU admission		COVID-19 deaths	
Risk group	Number of events	aHR (95% CI)	Number of events	aHR (95% CI)	Number of events	aHR (95% CI)
Use previous prescribed OCS as marker of history of an asthma attack*						
No asthma	20678	1	2198	1	7561	1
Asthma with 0 course of OCS	2827	1.15 (1.11-1.21)	297	1.11 (0.97-1.27)	732	1.03 (0.91-1.15)
Asthma with 1 course of OCS	1817	1.30 (1.23-1.37)	171	1.23 (1.03-1.48)	568	0.98 (0.86-1.12)
Asthma with 2 courses of OCS	814	1.37 (1.26-1.48)	88	1.61 (1.25-2.07)	268	1.12 (0.93-1.35)
Asthma with ≥3 courses of OCS	2553	1.54 (1.46-1.61)	225	1.89 (1.6-2.22)	990	1.39 (1.25-1.54)
Use prior hospitalisation for asthma as marker of history of an asthma attack*						
No asthma	23845	1	2463	1	8909	1
Asthma without prior hospitalisation	4643	1.24 (1.2-1.29)	498	1.26 (1.13-1.4)	1178	0.99 (0.9-1.09)
Asthma with prior hospitalisation	201	3.01 (2.59-3.49)	18	3.48 (2.16-5.6)	32	1.84 (1.13-3.02)

aHR: adjusted hazard ratio. CI: confidence interval. ICU: intensive care unit. OCS: oral corticosteroid. Hazard ratios were derived using cox proportional hazard model adjusting for age, sex, socioeconomic status, body mass index, number of risk groups of interest, number of non-asthma related hospitalisations within the two-year period prior to March 1, 2020 and vaccine status. * Two-year look back on all markers of history of an asthma attack was from March 1, 2020. ICU/deaths referred to those who had COVID-19 related ICU admissions or COVID-19 related death with or without previous ICU admissions. 42% of the cohort had missing ethnicity so this variable was not adjusted for in the Cox model. 1% of the cohort had missing SIMD or BMI and this 1% were excluded in the adjusted Cox model.

Table S8: Hazard ratio for COVID-19 hospitalisation, ICU admission and deaths comparing those with different markers of history of an asthma attack (defined in the one year prior to March 1, 2020) and those with no asthma in adults

	COVID-19 hospitalisation		COVID-19 ICU/deaths		COVID-19 deaths	
Risk group	Number of events	aHR (95% CI)	Number of events	aHR (95% CI)	Number of events	aHR (95% CI)
Use previous prescribed OCS as marker of history of an asthma attack*						
No asthma	21487	1	9554	1	7875	1
Asthma with 0 course of OCS	3343	1.19 (1.14-1.24)	1107	1.06 (0.97-1.15)	845	1 (0.9-1.12)
Asthma with 1 course of OCS	1464	1.33 (1.26-1.41)	535	0.98 (0.86-1.12)	450	0.94 (0.8-1.09)
Asthma with 2 courses of OCS	634	1.41 (1.29-1.54)	266	1.38 (1.17-1.64)	216	1.2 (0.97-1.47)
Asthma with ≥3 courses of OCS	1761	1.67 (1.58-1.76)	831	1.7 (1.53-1.88)	733	1.65 (1.47-1.86)
Use prior hospitalisation for asthma as marker of history of an asthma attack*						
No asthma	23849	1	10690	1	8910	1
Asthma without prior hospitalisation	4717	1.26 (1.21-1.3)	1578	1.12 (1.04-1.21)	1193	1 (0.91-1.1)
Asthma with prior hospitalisation	123	3.19 (2.63-3.86)	25	2.12 (1.3-3.46)	16	1.58 (0.78-3.22)

aHR: adjusted hazard ratio. CI: confidence interval. ICU: intensive care unit. OCS: oral corticosteroid. Hazard ratios were derived using cox proportional hazard model adjusting for age, sex, socioeconomic status, body mass index, number of risk groups of interest, number of non-asthma related hospitalisations within the two-year period prior to March 1, 2020 and vaccine status. * One-year look back on all markers of history of an asthma attack was from March 1, 2020. ICU/deaths referred to those who had COVID-19 related ICU admissions or COVID-19 related death with or without previous ICU admissions. 42% of the cohort had missing ethnicity so this variable was not adjusted for in the Cox model. 1% of the cohort had missing SIMD or BMI and this 1% were excluded in the adjusted Cox model.

Table S9: Hazard ratio for COVID-19 hospitalisation, ICU admission and deaths comparing those with different markers of history of an asthma attack and those with no asthma following positive COVID-19 test

	COVID-19 hospitalisation		COVID-19 ICU/deaths		COVID-19 deaths	
Risk group	Number of events	aHR (95% CI)	Number of events	aHR (95% CI)	Number of events	aHR (95% CI)
Use previous prescribed OCS as marker of history of an asthma attack*						
No asthma	19439	1	8711	1	7162	1
Asthma with 0 course of OCS	3674	1.09 (1.04-1.15)	1476	1.01 (0.91-1.11)	1260	1.03 (0.91-1.17)
Asthma with 1 course of OCS	2384	1.35 (1.29-1.42)	831	1.3 (1.18-1.43)	648	1.29 (1.15-1.45)
Asthma with 2 courses of OCS	2182	1.5 (1.4-1.61)	873	1.36 (1.18-1.56)	724	1.27 (1.07-1.5)
Asthma with ≥3 courses of OCS	1010	1.87 (1.8-1.95)	402	1.6 (1.48-1.74)	325	1.53 (1.4-1.68)
Use prior hospitalisation for asthma as marker of history of an asthma attack*						
No asthma	23838	1	10686	1	8906	1
Asthma without prior hospitalisation	4630	1.2 (1.16-1.25)	1557	1.06 (0.98-1.14)	1178	0.97 (0.88-1.07)
Asthma with prior hospitalisation	221	3 (2.61-3.46)	50	1.78 (1.26-2.51)	35	1.53 (0.97-2.42)

aHR: adjusted hazard ratio. CI: confidence interval. ICU: intensive care unit. OCS: oral corticosteroid. Hazard ratios were derived using cox proportional hazard model adjusting for age, sex, socioeconomic status, body mass index, number of risk groups of interest, number of non-asthma related hospitalisations within the two-year period prior to March 1, 2020, the time from March 1, 2020 to the date of infection (this is to adjust for different waves/dominant variants at baseline) and vaccine status. The vaccine status was measured at the date of positive COVID-19 test. * Two-year look back on all markers of history of an asthma attack was from the date of positive COVID-19 test. ICU/deaths include those had COVID-19 related ICU admissions or COVID-19 related death with or without previous ICU admissions.

Table S10: Hazard ratio for COVID-19 hospitalisation comparing those with different markers of history of an asthma attack (defined in the two years prior to March 1, 2020) and those with no asthma stratifying by the COPD status

	No COPD		With COPD	
	COVID-19 hospitalisation		COVID-19 hospitalisation	
Risk group	Number of events	aHR (95% CI)	Number of events	aHR (95% CI)
Use previous prescribed OCS as marker of history of an asthma attack*				
No asthma	19316	1	1222	1
Asthma with 0 course of OCS	2509	1.17 (1.12-1.22)	304	0.99 (0.86-1.14)
Asthma with 1 course of OCS	1331	1.38 (1.29-1.46)	479	1.07 (0.95-1.21)
Asthma with 2 courses of OCS	519	1.49 (1.36-1.64)	293	1.15 (0.99-1.33)
Asthma with ≥3 courses of OCS	1442	1.64 (1.54-1.74)	1089	1.39 (1.27-1.53)
Use prior hospitalisation for asthma as marker of history of an asthma attack*				
No asthma	21200	1	2482	1
Asthma without prior hospitalisation	3746	1.29 (1.24-1.34)	876	1.02 (0.93-1.11)
Asthma with prior hospitalisation	171	3.41 (2.9-4)	29	1.7 (1.13-2.54)

aHR: adjusted hazard ratio. CI: confidence interval. OCS: oral corticosteroid. Hazard ratios were derived using cox proportional hazard model adjusting for age, sex, socioeconomic status, body mass index, number of risk groups of interest, number of non-asthma related hospitalisations within the two-year period prior to March 1, 2020 and vaccine status. * Two-year look back on all markers of history of an asthma attack was from March 1, 2020. The interaction test between COPD and severity maker defined by prior asthma related hospitalisation had p value as <0.0001. The interaction test between COPD and severity maker defined by OCS use had p value as <0.0001.

The aHR of COVID-19 hospitalisation comparing those with a history of asthma attack vs those with no asthma is higher in the subset with no co-diagnosed of COPD compared to the subset with co-diagnosed COPD. This is because the reference group in the subset with COPD was a sicker population (those with COPD but with no asthma – the unadjusted HR of COPD was 4.45 (4.29-4.61) for COVID-19 hospitalisation compared to no COPD – shown in Table S12) while the reference group in the subset without COPD was those with neither asthma nor COPD. Our results show that for the group without COPD, asthma had a stronger impact on COVID-19 hospitalisation compared to the group with COPD.

Table S11: Hazard ratio for COVID-19 hospitalisation, ICU admission and deaths comparing those with different markers of history of an asthma attack (defined in the two years prior to March 1, 2020) and those with no asthma in the adults < 50 years old

	COVID-19 hospitalisation		COVID-19 ICU/deaths	
Risk group	Number of events	aHR (95% CI)	Number of events	aHR (95% CI)
Use previous prescribed OCS as marker of history of an asthma attack*				
No asthma	4597	1	614	1
Asthma with 0 course of OCS	837	1.15 (1.07-1.25)	120	1.25 (1.01-1.54)
Asthma with 1 course of OCS	340	1.75 (1.56-1.97)	43	1.6 (1.14-2.24)
Asthma with 2 courses of OCS	124	1.83 (1.52-2.2)	14	1.33 (0.72-2.42)
Asthma with ≥3 courses of OCS	286	2.53 (2.22-2.89)	69	4.15 (3.07-5.6)
Use prior hospitalisation for asthma as marker of history of an asthma attack*				
No asthma	4896	1	666	1
Asthma without prior hospitalisation	1216	1.3 (1.21-1.38)	181	1.42 (1.19-1.69)
Asthma with prior hospitalisation	72	4.12 (3.22-5.27)	13	6.13 (3.43-10.97)

aHR: adjusted hazard ratio. CI: confidence interval. ICU: intensive care unit. OCS: oral corticosteroid. Hazard ratios were derived using cox proportional hazard model adjusting for age, sex, socioeconomic status, body mass index, number of risk groups of interest, number of non-asthma related hospitalisations within the two-year period prior to March 1, 2020 and vaccine status. * Two-year look back on all markers of history of an asthma attack was from March 1, 2020. ICU/deaths include those had COVID-19 related ICU admissions or COVID-19 related death with or without previous ICU admissions.

The aHR of severe COVID-19 outcomes comparing those with a history of asthma attack vs those with no asthma is higher among those <50 years old compared to the general population. This is because the reference group in the < 50 years old was at lower risk for severe COVID-19 outcomes (Figure S2) compared to the general population. Our results show that for the <50 years old group, asthma had a stronger impact on severe COVID-19 outcomes compared to the general population.

Table S12: Hazard ratio for COVID-19 hospitalisation comparing those with different markers of history of an asthma attack (defined in the two years prior to March 1, 2020) and those with no asthma in adults – additionally adjusted for smoking status other than the risk factors in the main analysis

		COVID-19 hospitalisation	COVID-19 hospitalisation
Variable	Level	Adjusted HR (95% CI)	Adjusted HR (95% CI)
Age	linear	1.03 (1.03-1.03)	1.03 (1.03-1.03)
	nonlinear	NA	NA
Sex	Female	1	1
	Male	1.11 (1.08-1.14)	1.12 (1.09-1.15)
SIMD	1 - High	1	1
	2	0.79 (0.76-0.82)	0.79 (0.76-0.82)
	3	0.59 (0.56-0.61)	0.59 (0.56-0.61)
	4	0.54 (0.51-0.56)	0.54 (0.52-0.56)
	5-Low	0.5 (0.48-0.53)	0.51 (0.48-0.53)
Prior hospitalisation	0	1	1
	1	1.62 (1.56-1.67)	1.6 (1.54-1.66)
	2	1.93 (1.85-2.02)	1.89 (1.81-1.98)
	3+	2.94 (2.83-3.06)	2.82 (2.71-2.94)
Number of risk groups	0	1	1
	1	1.67 (1.61-1.73)	1.67 (1.61-1.73)
	2	2.5 (2.39-2.61)	2.47 (2.37-2.58)
	3	3.13 (2.98-3.3)	3.07 (2.92-3.23)
	4	3.65 (3.43-3.88)	3.55 (3.34-3.77)
	5+	4.65 (4.36-4.96)	4.46 (4.18-4.76)
BMI (kg/m2)	<18.5	1	1
	18.5-24.99	0.78 (0.71-0.87)	0.8 (0.72-0.88)
	25-29.99	0.84 (0.76-0.93)	0.86 (0.78-0.95)
	30-34.99	1.09 (0.99-1.21)	1.12 (1.01-1.24)
	≥35	1.37 (1.24-1.52)	1.4 (1.27-1.55)
Vaccine status	Unvaccinated	1	1
	0-27 days post 1st dose	0.69 (0.65-0.74)	0.69 (0.66-0.74)
	28+ post 1st dose	0.26 (0.24-0.29)	0.26 (0.24-0.29)
	0-27 post 2nd dose	0.22 (0.19-0.25)	0.22 (0.19-0.25)
	28+ post 2nd dose	0.15 (0.14-0.16)	0.15 (0.14-0.16)
Smoking status	Never	1	1
	Ex smoker	1.02 (0.98-1.05)	1.01 (0.97-1.04)
	Current smoker	0.88 (0.85-0.91)	0.85 (0.82-0.88)
	Unknown	0.61 (0.58-0.65)	0.6 (0.57-0.63)
Prior hospitalisation for asthma*	No asthma	1	-
	Asthma without prior hospitalisation	1.23 (1.18-1.27)	-
	Asthma with prior hospitalisation	2.98 (2.57-3.46)	-
Prior OCS prescriptions*	No asthma	-	1
	Asthma with 0 course of OCS	-	1.14 (1.09-1.19)

	Asthma with 1 course of OCS	-	1.32 (1.25-1.4)
	Asthma with 2 courses of OCS	-	1.41 (1.3-1.52)
	Asthma with ≥3 courses of OCS	-	1.59 (1.51-1.66)

aHR: adjusted hazard ratio. CI: confidence interval. OCS: oral corticosteroid. Hazard ratios were derived using cox proportional hazard model adjusting for age, sex, socioeconomic status, body mass index, number of risk groups of interest, number of non-asthma related hospitalisations within the two-year period prior to March 1, 2020 and vaccine status and smoking status. * Two-year look back on both markers of history of an asthma attack was from March 1, 2020. 42% of the cohort had missing ethnicity so this variable was not adjusted for in the Cox model. 1% of the cohort had missing SIMD or BMI and this 1% were excluded in the adjusted Cox model.

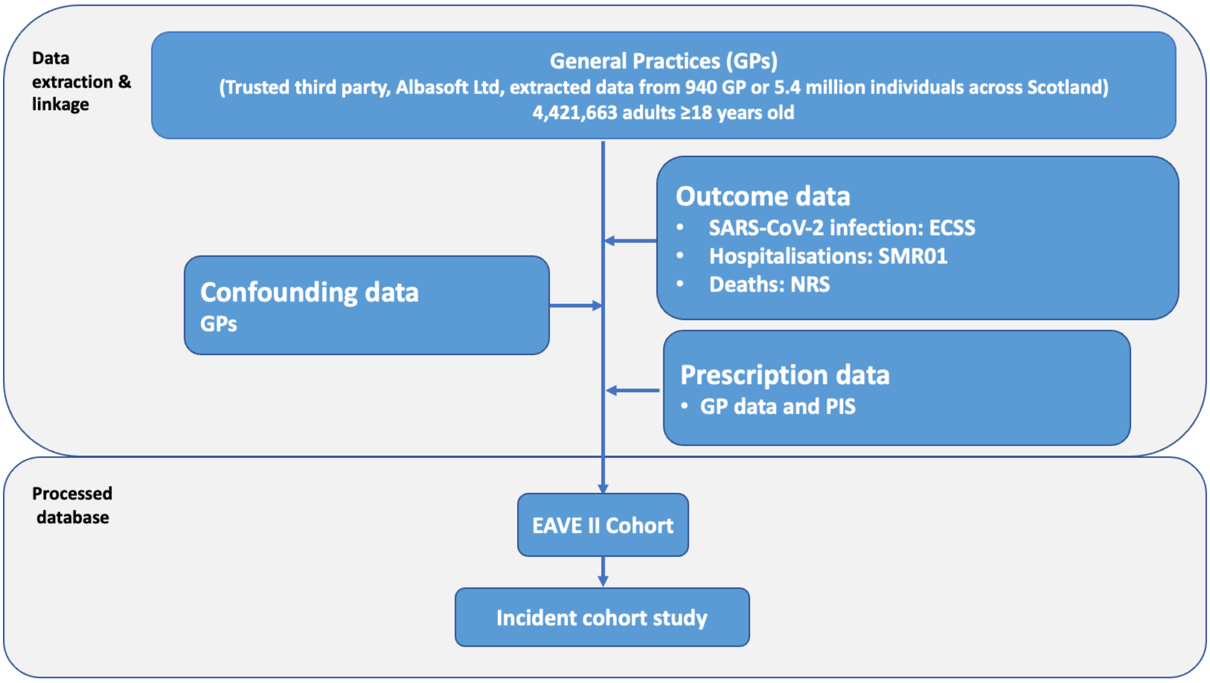

Community Health Index (CHI) numbers were used to link all datasets. ECSS: Electronic Communication of Surveillance in Scotland. SMR: Scottish Morbidity Record. NRS: National Records of Scotland. PIS: Prescribing Information System.

Figure S1: Data linkage diagram

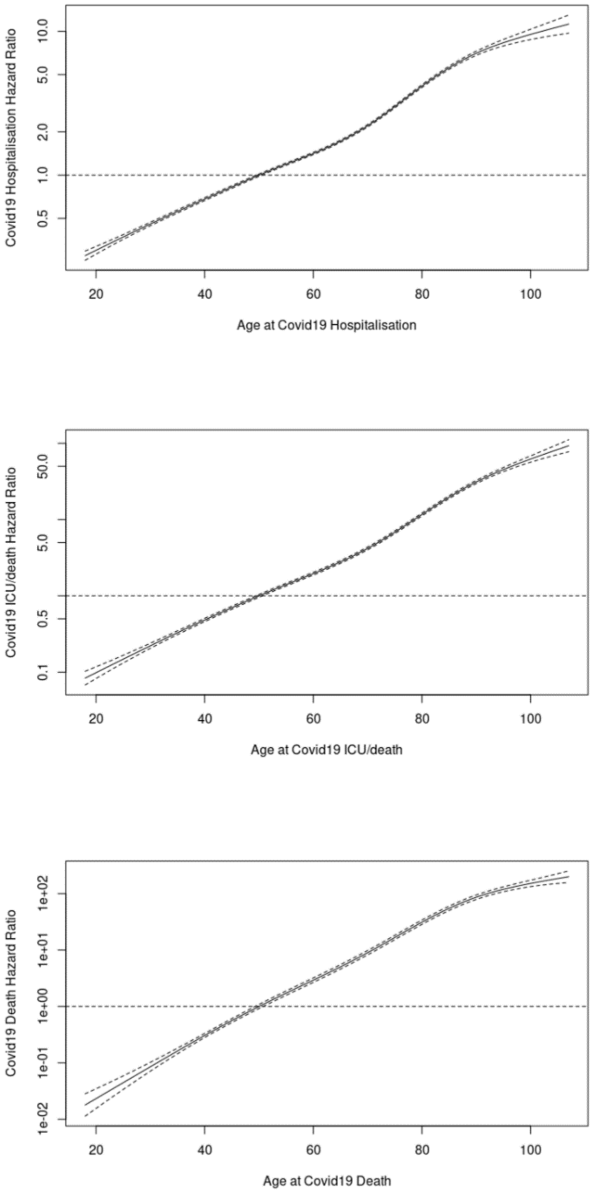

Figure S2: unadjusted non-linear age effect on COVID-19 hospitalisation, ICU admissions and death. Penalised splines were used in the statistical modelling.
